# Exergy-Based Analysis and Optimization of an Integrated Solar Combined-Cycle Power Plant

**DOI:** 10.3390/e22060655

**Published:** 2020-06-13

**Authors:** Louay Elmorsy, Tatiana Morosuk, George Tsatsaronis

**Affiliations:** 1 Energy Engineering Department, Campus El Gouna, Technische Universität Berlin, Ackerstraße 76, 13355 Berlin, Germany; louay.elmorsy@tu-berlin.de; 2 Institute for Energy Engineering, Technische Universität Berlin, Marchstraße 18, 10587 Berlin, Germany; georgios.tsatsaronis@tu-berlin.de

**Keywords:** integrated solar combined-cycle, linear fresnel collectors, direct steam generation, exergoeconomic optimization

## Abstract

The transition towards higher shares of electricity generation from renewable energy sources is shown to be significantly slower in developing countries with low-cost fossil fuel resources. Integrating conventional power plants with concentrated solar power may facilitate the transition towards a more sustainable power production. In this paper, a novel natural gas-fired integrated solar combined-cycle power plant was proposed, evaluated, and optimized with exergy-based methods. The proposed system utilizes the advantages of combined-cycle power plants, direct steam generation, and linear Fresnel collectors to provide 475 MW baseload power in Aswan, Egypt. The proposed system is found to reach exergetic efficiencies of 50.7% and 58.1% for day and night operations, respectively. In economic analysis, a weighted average levelized cost of electricity of 40.0 $/MWh based on the number of day and night operation hours is identified. In exergoeconomic analysis, the costs of thermodynamic inefficiencies were identified and compared to the component cost rates. Different measures for component cost reduction and performance enhancement were identified and applied. Using iterative exergoeconomic optimization, the levelized cost of electricity is reduced to a weighted average of 39.2 $/MWh and a specific investment cost of 1088 $/kW. Finally, the proposed system is found to be competitive with existing integrated solar combined-cycle plants, while allowing a significantly higher solar share of 17% of the installed capacity.

## 1. Introduction

It is a fact that the global energy sector is in a transitional phase towards a higher share of renewable energy supply. Many countries, in particular in the Middle East and North Africa (MENA) region, have decent potential for deploying concentrated solar power (CSP) with a direct normal irradiance (DNI) of up to 2500 kWh/m^2^a, but face many challenges in introducing such systems owing to their high capital investment cost, technological advancement, as well as lack of supporting regulations and financial incentives. Solar energy technologies without storage cannot compete with conventional baseload plants owing to their intermittent nature. In many countries in the MENA region, such as Egypt, the operation policy of the existing thermal power plants is based on considering natural gas as the primary fuel owing to its evident economic and environmental advantages, representing more than 80% of the installed capacity in the past years [1].

Thermal energy conversion enables CSP plants the great advantage to offer dispatching power and increase the system capacity factor when integrated with either thermal energy storage or conventional fuels [2,3]. To provide reasonable storage durations, the integration of thermal energy storage (TES) necessitates the oversizing of the solar field, which implies an even bigger capital investment associated with TES and the solar field. The integration of CSP with fossil fuels paves the way for development and investment in CSP technology at a moderate marginal cost. This can lead to cost regression of the technology and expedite the transition towards pure concentrated solar power plants in the region, for example, through local manufacturing. Both Egypt and Morocco were identified to offer the highest manufacturing attractiveness for CSP components in the MENA region [4,5]. In such hybrid plants, solar energy reduces the fossil fuel consumption, until pure CSP becomes more cost competitive and large storage capacities are well established and their investment costs decrease. A significant increase in the solar plants’ share in total electricity generation can then take place.

These hybridized systems can be referred to as integrated solar combined-cycle (ISCC) power plants. ISCC power plants represent the most efficient integration of fossil and solar resources for baseload power generation [6]. Natural gas-fired combined-cycle power plants (NG-CCPPs) are widely known to reach the highest efficiencies of up to 63%, as reported for HL-Class Siemens AG turbine [7]. Although NG-CCPPs operate by fossil fuel combustion, they are considered as the cleanest conventional power generation technology. Apart from natural gas, an ISCC plant has a second source of thermal energy, which is represented by the solar field. ISCC plants have a similar arrangement of components as regular NG-CCPPs. Yet, the solar field technology and location could differ, and depend on the assigned purpose: as economizers, evaporators, and/or superheaters of the steam cycle.

Linear Fresnel collectors (LFCs) are less expensive compared with parabolic trough collectors (PTCs), and offer maturity especially with direct steam generation (DSG) [8]. DSG is a technology receiving much attention from the research community [9]. DSG refers to steam generation inside the solar field, thus avoiding additional heat exchangers between the heat transfer fluid (HTF) and steam cycle, accordingly reducing the power plant configuration complexity and avoiding additional irreversibilities. DSG reaches higher temperatures that are difficult to achieve using synthetic oil, thus the overall plant efficiency can be higher. From the economic viewpoint, beside cost reductions attributed to using less heat exchangers, it also means further cost reduction by avoiding the use of expensive HTFs as synthetic oil. Operation and maintenance costs are also lower than those of synthetic oil-based plants as auxiliary heating systems are not needed. Finally, from the environmental viewpoint, major environmental hazards such as leakage and fire of HTF are avoided [9,10,11].

In particular, LFC technology integration with NG-CCPPs may enable ISCC plants to reach competitive costs and offer a more environmentally friendly solution as baseload power plants for developing countries with significant natural gas and solar resources (for example, Egypt).

## 2. Literature Review

As ISCC power plants use the advantages of both the currently most efficient energy conversion system and the renewable energy conversion technology with the highest potential capacity factor (concentrated solar power), the technology is considered to be a cost-effective baseload alternative to expedite the transition from conventional energy generation to a more sustainable generation. In recent years, great interest has arisen in quantifying the advantages of ISCC systems.

Alqahtani and Echeverri [12] conducted a study on ISCC technology comparing five different locations in the USA with different solar irradiance, temperatures, NG prices, capacity factors, tax incentives, and capital costs. The study showed that integrating CSP with NG-CCPP reduces the levelized cost of electricity (LCOE) by 35–40% if compared with standalone CSP, also taking dispatchability into account. The analysis also showed that ISCC has more economic advantages in harnessing solar energy than standalone CSP with or without thermal energy storage. However, if ISCC is compared to NG-CCPP under the current NG prices, “carbon prices”, or high subsidies, CCPP would produce electricity at a lower cost.

Achieving higher capacity factors in solar plants or capital investment cost reductions of concentrated solar power components will favor the ISCC [12]. Nowadays, great attention is paid to developing NG-fired ISCC plants utilizing DSG technology. This technology is expected to lead not only to low cost electricity, but also process heating [13]. Nezammahalleh et al. [14] investigated three different types of CSP plants, including two ISCC plants using DSG and another using an HTF, and showed that the DSG-ISCC plant produces electricity at a 2.4% lower cost than that of the HTF-ISCC plant owing to the lower investment cost and higher efficiency. The study also showed that DSG-ISCC plants consume less fuel than HTF-ISCC plants, consequently saving more money and producing less CO_2_ emissions. The study suggests that DSG-ISCC is the best option for arid countries that are rich in natural gas resources [14].

Rovira et al. [15] presented the research on different configurations of ISCCs. Solar integration was considered using both HTF and DSG technologies. Each technology comprised four different arrangements for the solar heat integration in the CCPP—the different arrangements included utilizing the solar heat for (a) preheating and evaporation; (b) only evaporation; (c) evaporation and superheating; and lastly (d) preheating, evaporation, and superheating. The authors emphasized the importance of using the solar field for steam generation. The study concluded that the DSG-ISCC is the better option and strongly penalized the HTF-ISCC as it required an additional steam generator [15]. A limitation of the study is the consideration of only the PTCs, without considering other CSP technologies.

Before the implementation of Kuraymat in 2004—the only ISCC plant in Egypt—Horn et al. [16] conducted a feasibility study to select the suitable ISCC configuration under given local conditions. The study compared two arrangements of ISCC, PTC with HTF and solar tower technology with air as working fluid, with the former having solar input of 90 MW_th_ and the latter 80 MW_th_. Both plants were designed having 127 MW capacity. The study concluded that the PTC-ISCC results in similar LCOE than that of the air solar tower (29–38 $_2018_/kWh), but produces 200 tons less CO_2_ per year [16]. The study did not consider other CSP technologies.

A number of utility-scale ISCC projects exist in the world, see Table 1. With exception of the Dadri project, which utilizes LFC technology, all reviewed ISCC plants (operating and under construction) use PTCs and HTF.

A comparative life cycle assessment of the four types of CSP plants was presented by Kuenlin et al. [22]. The study concluded that the PTC plant is the one with the worst environmental performance among the considered CSP technologies as a result of using the synthetic oil as the network HTF and the molten salt storage system. With the aid of direct steam generation, the utilization of a hazardous HTF can be avoided. One of the most important features of the DSG technology is operating the plant at a higher temperature and pressure of the fluid inside the collector tubes without the limitations of using a heat transfer oil [23]. DSG, in the commonly used line-focusing solar collector technology PTC, was shown to be technically challenging, for example, owing to the constant motion of the whole collector (mirrors and receiver) and the high operating temperatures and pressures, meaning that the receiver tubes and interconnection between collectors are very critical components that still require experimental validation [24]. Thanks to its static design, linear Fresnel collector technology is not only cheaper, less technically challenging, and easier to maintain [25], but also more mature with DSG. According to a study conducted by the National Renewable Energy Laboratory (NREL), there is a growing interest in LFC using DSG to utilities in the united states as a candidate for integration with CCPP [26]. Moreover, the use of LFC was emphasized to be particularly relevant for ISCC systems [12,13].

A single-objective exergoeconomic optimization using genetic algorithm applied to an ISCC (having the same configuration as Yazd power plant in Iran) was presented by Baghernejad and Yaghoubi [27]. The power plant works with PTC that uses Therminol VP-1 as HTC. The objective function, which is the cost of product, decreased by 11% from 58.8 to 53.0 $/MWh at the expense of an increase of 13.3% in the capital investment. The exergetic efficiency also increased from 43.8% to 46.8% [27]. The same authors published another optimization study for the same ISCC power plant using a multi-objective approach to eliminate the shortcomings of the first study by satisfying both the exergetic and economic objectives [28]. The findings are as follows: increase in the exergetic efficiency by 3.2% and decrease in the cost of the product by 3.8% [29].

The reviewed literature confirmed that ISCC merges the advantages offered by both NG-CCPPs and CSP plants. A number of system configurations, solar collector technologies, working fluids, and operation ranges were discussed and analyzed. Yet, no optimal system configuration could be derived from the reviewed literature. Taking into account the identified economic edge of LFC technology over other CSP technologies, as well as its maturity with DSG, enabling higher efficiencies and minimizing environmental risks, the presented research aims to identify an optimal ISCC system configuration. An optimized ISCC configuration making use of the advantages of LFC and DSG could reach cost competitiveness in the MENA region. In this paper, a special ISCC configuration utilizing LFC and DSG is presented and analyzed using exergetic, economic, and exergoeconomic analysis. An optimized system is derived with the help of a single-objective iterative exergoeconomic optimization.

## 3. Methodology

In this paper, the design, analysis, and optimization of an integrated solar combined-cycle power plant is presented. The proposed system design was simulated using software with industrial application. The results obtained in the simulation were further analyzed using exergy, economic and exergoeconomic analysis. On the basis of the results, a single-objective iterative and knowledge-based exergoeconomic optimization was applied, aiming to minimize the ISCC systems LCOE.

### 3.1. Design and Simulation

**Figure 1:**
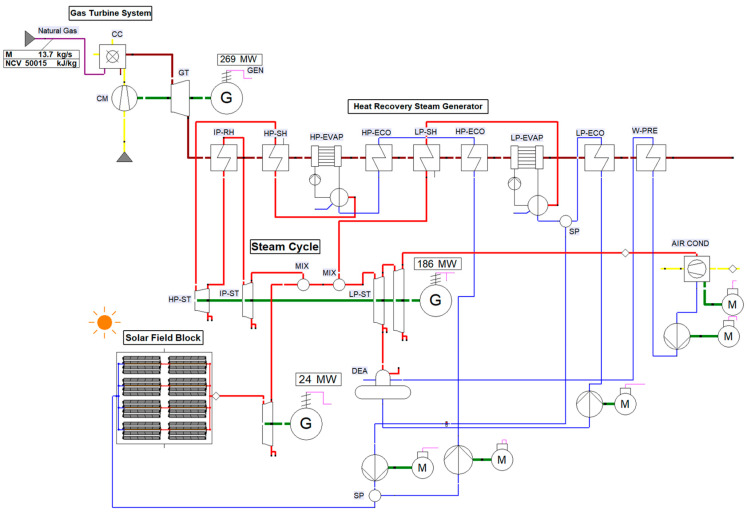
Schematic of the integrated solar combined-cycle (ISCC) using EBSILON^®^Professional.

The proposed configuration of the base case ISCC system is based on the parallel connection between the solar field and the gas turbine as shown in Figure 1. The high pressure superheated steam is partially generated by the high-temperature gas turbine exhaust in the heat recovery steam generator (HRSG) and partially inside the solar field during day operation. However, during night operation in the absence of solar radiation, steam is generated as a result of only the gas-turbine exhaust gases.

The installed capacity ($P_{inst}$) of the ISCC plant was selected to be 475 MW based on the gas turbine SGT5-4000F from Siemens AG [30]. The gas turbine system is the largest source of electricity in the ISCC. The compression ratio of the gas turbine system is 18.0:1 and 20.1:1 for day and night operations, respectively. The air to fuel ratio (λ = 2.516) is regulated by a controller to achieve a constant gas expander outlet temperature of 600 °C during both operations. Consequently, an adiabatic temperature of the combustion process of 1287 °C and 1334 °C are achieved for both day and night operations, respectively.

The steam turbine block is the second largest source of electricity production in the ISCC. The Siemens AG steam turbine SST-3000 is selected, which covers the power output range from 90 to 250 MW with steam condition of up to 177 bar and 565 °C and reheat of up to 610 °C [30]. In the proposed ISCC design, the steam turbine operates with high, intermediate, and low pressure levels, which operate at 170 bar, 80 bar, and 9.5 bar, respectively, during night operation, and 156.8 bar, 74 bar, and 15 bar, respectively, during day operation.

Direct steam generation was selected as the solar thermal conversion method, using water (steam) as the working fluid of the power cycle. This selection was based on a number of advantages:

- Achieving higher temperatures and pressures than with common HTF;
- Despite higher thermal losses in the solar field, the lack of a secondary working fluid improves the efficiency of the power cycle [24,31].

The LFC consists of vacuum tube absorbers and is simulated as a once-through system where the preheated water is economized, evaporated, and superheated inside the collector tubes. The steam is produced at 50 bar and 500 °C.

For reducing part-load losses that would result in a drop in the efficiency of the high- and intermediate-pressure (HP and IP) turbines during absence of solar heat, a separate solar steam turbine was allocated to expand the superheated stream at 50 bar provided by the LFC and bypass the low-pressure (LP) and IP turbines. The exiting steam to be directly fed at 15 bar to the low-pressure (LP) turbine. The Siemens AG steam turbine SST-300 size 40 was selected. The solar turbine is suitably designed for CSP applications allowing short start-up times and quick load changes, and guaranteeing high efficiency over a wide range of operation modes. The turbine is also suitable for DSG applications [32]. The solar steam turbine output power is up to 25 MW and the steam inlet pressure is up to 140 bar [33]. During day operation, the turbine output power is 24 MW as a result of the steam supplied to it from the solar field. However, the solar share is equivalent to 17% of the total plant installed capacity (representing 81 MW after accounting for the steam being fed to the LP turbine) under full-load operation at design point direct normal irradiance (DNI) of 850 W/m^2^.

The highest performance is reached by ISCCs configurations that reduce the irreversibilities in the HRSG [15]. Hence, special attention is paid during the design phase for making use of the exergy of the exhaust gases in the HRSG. During both day and night operations, the exhaust gas temperatures to the environment are 98 °C and 106 °C, respectively.

Critics of solar plants state that the technology is not sustainable as they consume large amounts of water, although they are installed in desert regions that do not have such resources. Moreover, wet-cooled plants are more efficient than dry-cooled plants [34]. However, dry cooling of the condenser can reduce water consumption by up to 95% [35,36]. Depending on the availability of water at any site, the condenser is either dry or wet cooled.

The proposed plant location is the city of Aswan, Egypt. Although the city is located on the river Nile, an air-cooled condenser (ACC) was simulated to investigate its performance in the ISCC as water is highly variable, limited, and becoming a major challenge facing Egypt [37]. The mass flow rate of the cooling air is regulated by a controller to keep the temperature at the outlet of the condenser below 40 °C.

To have a constant output power of 475 MW, the fuel consumption reaches 13.7 kg/s during the day operation and reaches its maximum of 15.8 kg/s during the night operation. The NG lower heating value (LHV) is assumed as 50,015 kJ/kg. The contribution of the solar thermal energy ($Q_{sol}$= 243 MW_th_) in the total heating load is measured by its share in the system’s total heat input ($Q_{f}+Q_{sol}$) and is calculated as 26%, as defined in Equation (1) [38].

(1) $$X_{sol}=\frac{{\dot{Q}}_{sol}}{{\dot{Q}}_{f}+{\dot{Q}}_{sol}}=\frac{{\dot{Q}}_{sol}}{m_{f}\cdot LHV+{\dot{Q}}_{sol}}.$$

The ratio between the effective solar thermal energy that is transferred to the working fluid ($Q_{eff}$ = 205 MW_th_) and the heat of the gas turbine system (after the combustion process) is approximately 21%. The ratio between the effective thermal energy from the solar field and the power plant net electrical output is around 43%.

The ISCC power plant was modelled with the help of the simulation program EBSILON^®^Professional version 14.02 [39]. The modelling of the steam cycle is based on the IAPWS1-IF-97 (International Association for the Properties of Water and Steam) properties, while for the exhaust gases, the ideal gas properties without real gas corrections are used. The configuration was simulated in day and night operation modes under steady-state conditions. The night operation is considered as the reference design case for the simulation as the gas turbine system, which provides around two-thirds of the total system capacity, is working at its full-load. The day operation is a sub-profile from the night operation. A relative humidity of 34.8% is used. The main differences between day and night operations are the contribution of the solar field and the average ambient temperature of 27.4 °C and 13.0 °C, respectively [40]. The technical data used for simulation are summarized in Table 2.

### 3.2. Exergy Analysis

In the exergy analysis, the physical and chemical exergies were considered for calculating the total exergy of streams. The physical exergy ($e_{j}^{PH}$) was calculated according to Equation (2) and the values are extracted directly from EBSILON^®^Professional. Here, *h*, *T*, and *s*; denote the enthalpy, temperature, and entropy, respectively. Moreover, the subscripts j and 0 denote a given and reference state, respectively.

(2) $$e_{j}^{PH}=\left(h_{j}-h_{0}\right)-T_{0}\left(s_{j}-s_{0}\right).$$

For the calculation of the chemical exergies ($e_{j}^{CH}$), the model of Ahrendts [41] was used. In order to apply the exergy analysis on components and system level, the exergy of fuel (${Ė}_{F}$) and exergy of product (${Ė}_{P}$) approach was applied in accordance with Bejan et al. [42]. An exergy balance could be seen in Equation (3), which could be applied on both the components and system level, where (${Ė}_{D}$) denotes the exergy destruction and the exergy losses (${Ė}_{L}$) are only considered for the overall system.

(3) $${Ė}_{F}={Ė}_{P}+{Ė}_{D}+{Ė}_{L}.$$

The exergetic efficiency (ε) is the ratio between ${Ė}_{P}$ and ${Ė}_{F}$ for either the components or the whole system, as seen in Equation (4). The exergy of fuel for the proposed ISCC includes both the exergy supplied by the NG and the exergy of the solar heat. The solar exergy could be calculated using the simplified Equation (5) presented in [43]. $T_{sun}$ is the surface temperature of the sun (5679 K).

(4) $$\varepsilon =\frac{{Ė}_{P}}{{Ė}_{F}},$$

(5) $${Ė}_{sol}={\dot{Q}}_{sol}\left(1-\frac{4}{3}\frac{T_{0}}{T_{sun}}\right),$$

The air-cooled condenser (ACC) is considered as a dissipative component as exergy is only destroyed and transferred to the environment without obtaining a useful exergetic product for the component itself, but the component is essential for the thermodynamic cycle of the ISCC. Thus, no exergetic efficiency could be defined for the ACC.

After extracting the steams’ physical properties from EBSILON^®^Professional, the exergy analysis was conducted using the Visual Basic for Applications (VBA) tool embedded in Microsoft Excel. Exergy analysis was applied to the system separately for day and night operations. The analysis resulted in the overall system and components exergy destruction values and exergetic efficiencies, as well as other exergetic variables.

### 3.3. Economic Analysis

An economic analysis was conducted to estimate the total project expenditures. The total revenue requirement (TRR) method was applied [42]. The cost estimating charts presented in [44] were used to estimate the purchase costs of ISCC components. The specific investment cost of the LFC solar field was considered as 152 €/m^2^ according to [45]. The ACC was estimated with 120,000 $/MW_th_ based on a reference specific investment cost presented by [46].

The fixed capital investment (FCI) was estimated through the total components’ cost, offsite costs, and contingences. The offsite costs include service facilities as well as civil and architectural work, and were estimated as 12% of the total components’ cost. Contingencies were estimated as 10% of the components’ cost. Startup costs and working capital were estimated as 7% and 5% of the FCI, respectively. Allowance for funds used during construction (AFUDC) were estimated based on three installments over the period of 3 years—40%, 40%, and 20% of the FCI in addition to their interest. Summation of the FCI, startup costs, working capital, and AFUDC resulted in the total capital investment (TCI) of the ISCC.

Financing was assumed to be in $US with an inflation rate of 1.56%, which is a 10-year (2009–2018) average [47]. Moreover, a 10-year average value was used for the exchange rate between Euro and USD (1.28 €/USD). The interest rate consists of a base interest rate of 2%, which is the $US LIBOR plus 6% interest rate margin for the lenders, which is a slightly high owing to the use of the linear Fresnel technology that is considered still to be relatively new, summing up to the total interest rate of 8%. Other important economic assumptions are the following: natural gas price of 3 $/MMBtu, which is the current natural gas price in Egypt for electricity production purposes [48]; an average nominal escalation rate of 3%; plant capacity factor of 80%; and 20 years as the economic lifetime. The economic analysis resulted in, among others, the TRR, the specific investment cost of the plant, and the cost rate associated with the investment and the operation and maintenance cost (Ż), which will be used as input for the exergoeconomic analysis. Ż is either the components’ or total system cost rate, as shown in Equations (6) and (7). All costs in the economic analysis are in U.S. Dollars and reflected to the year 2018.

(6) $${Ż}_{k}={Ż}_{k}^{CI}+{Ż}_{k}^{OM}=\frac{CC_{L}}{\tau }\frac{CM_{k}}{\sum_{n}CM_{n}}+\frac{OMC_{L}}{\tau }\frac{CM_{k}}{\sum_{n}CM_{n}},$$

(7) $${Ż}_{tot}=\sum {Ż}_{k},$$

The levelized total revenue required ($TRR_{L}$) was estimated through the levelized carrying charges ($CC_{L}$), the levelized operation and maintenance costs ($OMC_{L}$), and the levelized fuel costs ($FC_{L}$); see Equation (8). The $CC_{L}$ was estimated through the TCI and the capital recovery factor, which is calculated through the interest rate and the economic lifetime. The $OMC_{L}$ was estimated based on 5% of the FCI and the general constant escalation levelization factor (CELF), which is calculated through the inflation rate, the interest rate, and the economic lifetime. The $FC_{L}$ was estimated based on the above mentioned NG price and the fuel CELF, which is calculated with the same assumption as the general CELF except for the use of the NG escalation rate instead of the currency inflation rate.

(8) $$TRR_{L}=CC_{L}+OMC_{L}+FC_{L},$$

For estimating the corresponding LCOE for day and night operations separately, Equation (9) was used taking into account the percentages of 46% and 54% of the annual full load hours (FLHs), respectively.

(9) $$LCOE=\frac{TRR_{L}}{P_{inst}\cdot FLH}.\;$$

A weighted average LCOE between both operations was estimated using both operations’ LCOE and their corresponding operation shares.

### 3.4. Exergoeconomic Analysis and Optimization

When exergy and cost are considered for conducting a thermoeconomic analysis, the term thermoeconomic changes to exergoeconomic [42]. Exergoeconomic analysis was further applied to the evaluated ISCC design. The exergy costing principle was applied on both the system and components level, in accordance with [42]. With the help of the fuel and product principal presented in [49], the auxiliary equations were obtained.

The cost of each stream associated with its exergy rate was calculated according to Equation (10), where ${Ċ}_{j}$ is the cost rate of the j-th stream, ${Ė}_{j}$ is the exergy rate of the *j*-th stream, and $c_{j}$ is the specific cost per unit of exergy.

(10) $${Ċ}_{j}=c_{j}{Ė}_{j}.$$

Cost balancing equations were defined for all ISCC components as well as the whole system in accordance with Equation (11), where ${Ċ}_{P}$ refers to the cost of the product, ${Ċ}_{F}$ is the cost of fuel, and ${Ċ}_{L}$. is the cost of losses. ${Ċ}_{L}$. is only considered for the complete system and is calculated according to Equation (12), where $c_{F,tot}$ is the specific fuel cost of the ISCC.

(11) $${Ċ}_{P}={Ċ}_{F}+Ż-{Ċ}_{L},$$

(12) $${Ċ}_{L}=c_{F,tot}{Ė}_{L}.$$

The cost rate associated with the exergy destruction (${Ċ}_{D}$) for the components and the whole system was calculated as per Equation (13). Exception was made only for the solar field where the specific cost of product ($c_{P}$) was used instead of the specific cost of fuel ($c_{F}$).

(13) $${Ċ}_{D}=c_{F}{Ė}_{D}.$$

The LCOE was also calculated by means of the exergoeconomic analysis according to Equation (14). The LCOE resulting from the exergoeconomic analysis must be the same as that resulting from the economic analysis. The ${Ċ}_{ACC}$ is the cost difference of the ACC and should be charged to the LCOE as no product cost was calculated for such dissipative component, as no exergetic product was defined. However, the cost associated with the capital investment, operation and maintenance, and ${Ė}_{D}$ of the ACC should be included in the ISCC’s final product cost.

(14) $$LCOE=\frac{{Ċ}_{P}+{Ċ}_{L}+{Ċ}_{ACC}}{{Ė}_{P}}.$$

Other important factors such as the exergoeconomic factor $(f_{k}$) and the relative cost difference ($r_{k}$) were also obtained. The $f_{k}$ identify the major cost source, whether it is the cost rate related to the component investment and operation and maintenance or related to its exergy destruction as seen in Equation (15). However, the relative cost difference $r_{k}$ expresses the relative increase in the average cost per exergy unit between fuel ($c_{F,k}$) and product ($c_{P,k}$) of any component, as in Equation (16). Both are useful variables for evaluating and optimizing components in an iterative cost optimization of a system.

(15) $$f_{k}=\frac{{Ż}_{k}}{{Ż}_{k}+{Ė}_{D,k}},$$

(16) $$r_{k}=\frac{c_{P,k}-c_{F,k}}{c_{F,k}}.$$

The results of the exergoeconomic analysis were used to facilitate optimizing the ISCC. The exergoeconomic optimization concept was used, which is a “knowledge-based” single-objective optimization. The aim of the iterative exergoeconomic optimization is to maximize the cost effectiveness of the ISCC by minimizing the system’s LCOE. Components to be optimized are prioritized according to their sum of ${Ċ}_{D,k}+$ ${Ż}_{k}$. The corresponding exergoeconomic factor $f_{k}$ is an indication of either low $\varepsilon_{k}$ (high ${Ė}_{D,k}$) or high investment cost, and should be adjusted during the optimization phase to reach a reduced LCOE.

Before starting the iterative optimization, variables that have significant influence on the system thermodynamic performance and investment costs should be selected. Such variables are named decision variables.

## 4. Results

### 4.1. Base Case

**Figure 2:**
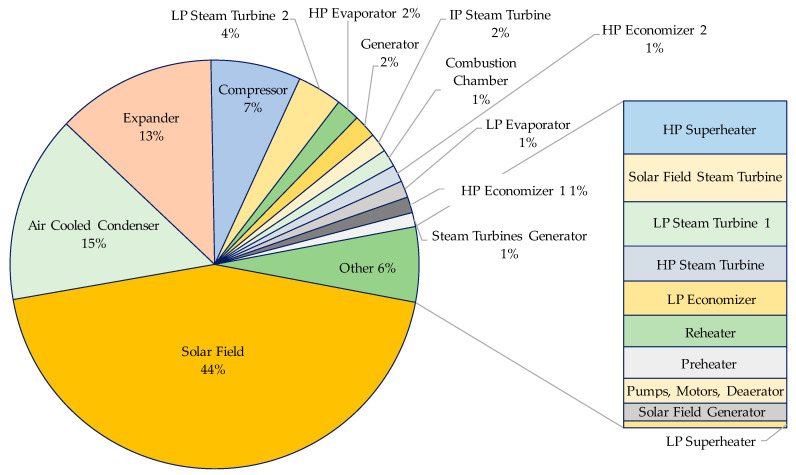
Component’s cost breakdown. LP, low-pressure; IP, intermediate-pressure; HP, high-pressure.

The exergy analysis results showed that the proposed ISCC base case reaches an exergetic efficiency of 50.7% during daytime, which increases during night operation to 58.1%. The components’ cost breakdown is shown in Figure 2. The four largest contributors to the total components purchased cost are the solar field, ACC, expander, and compressor, representing 44%, 15%, 13%, and 7%, respectively, of a total of approximately $325 million. The economic assessment resulted in a total capital investment of $518.3 million with a specific investment cost of 1091 $/kW_ex_ and a levelized total revenue required of $133 million. The proposed ISCC reached a LCOE of the base case design amounting to 38.2 $/MWh and 41.5 $/MWh during day and night operations, respectively, resulting in a weighted average of 40.0 $/MWh based on the number of day and night operation hours.

In order to optimize the system, the base case design was evaluated and the components were prioritized according to their cost-importance for optimization. The components are ranked according to their total cost rates (${Ċ}_{D,k}+$ ${Ż}_{k}$). The exergetic efficiencies $\varepsilon_{k}$, exergoeconomic factors $f_{k}$, and component cost rates of the six highest ranked components of the ISCC system are given in Table 3. On the basis of the necessary reduction in ${Ċ}_{D,k}$ or ${Ż}_{k}$, component parameters that should be changed were identified. The identified decision variables are listed in Table 4.

The *solar field* (SF) shows an exceptionally low exergetic efficiency of 41.3% and comes on top of components that should be optimized owing to the very high cost rate value (8251 $/h). The solar field contributes the highest ${Ċ}_{D}$ as well as ${Ż}_{k}$ among all components. Having low $f_{k}$ indicates the higher share of the component’s ${Ċ}_{D}$, thus an increase in the exergetic efficiency of the solar field is expected during optimization. Changing the size of the solar field could be the most effective measure to decrease the total cost rate of the component and increase the cost-effectiveness of the system. Yet, it was decided to keep the solar field size unchanged and, instead, only operating parameters of the solar field were manipulated in an attempt to increase its exergetic efficiency. The exergetic efficiency of the solar field improves with increased temperature and pressure of the steam entering the LFC, see Table 5.

The *combustion chamber* (CC) has the second highest cost rate among the components (3530 $/h) with a low exergetic efficiency of 71.5%. The low $f_{k}$ indicates the large contribution of the ${Ċ}_{D}$ and its relatively negligible ${Ż}_{k}$. Although the combustion process efficiency is limited owing large irreversibilities as a result of chemical reaction, heat transfer, mixing, and friction, a small reduction in its ${Ė}_{D}$ would yield a significant improvement in the system performance, as its ${Ė}_{D}$ contributes a significant share in the total system ${Ė}_{D}$. Hence, an improvement in its exergetic efficiency is recommended, which may be achieved by increasing both the compressor pressure ratio $\pi_{\mathrm{CM}}$ and the gas turbine inlet temperature TIT.

The *air-cooled condenser* results in high ${Ż}_{k}$ compared with its ${Ċ}_{D}$, resulting in a high $f_{k}$ (0.808). Thus, a reduction in investment cost of the ACC at the expense of a reduced exergetic efficiency would be accepted to improve the cost-competitiveness of the ISCC system. The ${Ż}_{ACC}$ could be reduced when changes are applied to the steam pressure $p_{SF}$ and the condenser pressure $p_{CD}$, see Table 4 and Table 5.

For the *gas turbine expander* (GT-EXP) and *compressor* (COMP), a reduction in the exergetic efficiency would also be tolerated to reduce the overall components’ cost rate and the LCOE of the system. Both components are influenced by the compressor pressure ratio $\pi_{\mathrm{CM}}$ (Table 4). Both the suggestion for the reduction in the $\pi_{\mathrm{CM}}$ and the TIT contradict the implications suggested by the other components for the same parameters, see Table 5.

The *low-pressure steam turbine 2* (LP-TUR-2) shows a relatively low $f_{k}$ (0.329) compared with the average typical values of turbomachinery of up to 0.75 [42,50], thus increasing its exergetic efficiency is an aim of the optimization. The performance of the LP steam turbine 2 is thereby influenced by a number of decision variables (${\dot{m}}_{SF}$, $p_{SF}$, $p_{IP-T}$, and $p_{CD}$).

### 4.2. First Iteration

In the first iteration of the ISCC, parametric optimization was performed and no structural optimization was implemented on the ISCC design. All suggested changes as well as the new operating parameters of the first iteration are shown in Table 5. The suggested changes are shown with arrows indicating either an increase (↑) or decrease (↓) of the respective parameter. In the case of contradicting objectives, the sum of the total cost rates associated with the respective suggestion is compared. The suggestion with the higher $({Ċ}_{D,k}+$ ${Ż}_{k})$ is implemented.

By decreasing the mass flow of the stream entering the LFC by 2.6%, the temperature at the outlet is elevated to 525 °C. As a result of the elevated pressure (55 bar) and the reduced logarithmic mean temperature difference (LMTD), the heat transfer is improved and the exergetic efficiency of the solar field is elevated by 0.5 percentage points to 41.8%.

For the combustion chamber, an increase in the compressor pressure ratio $\pi_{\mathrm{CM}}$ to 21.1 as well as an increase in the TIT to 1365 °C led to an increase of its exergetic efficiency to 71.9% and a decrease of the ${Ė}_{D}$ from 233.2 MW to 226.5 MW.

For reducing the ${Ż}_{k}$ of the condenser, the condenser pressure $p_{CD}$ was reduced to 0.08 bar, which resulted in a lower steam condensation temperature of 42.5 °C instead of 45.2 °C. The steam temperature is still higher than that of the average environmental temperature. As $p_{CD}$ reduces the difference between the environmental temperature and the condensation temperature decrease, thus requiring lower air mass flow for the condensation process to take place (18.7 ton/s instead of 19.2 ton/s), which results in a smaller equipment size, and consequently lower investment of $47.4 million.

Aiming for an increase in the exergetic efficiency of the LP steam turbine 2, $p_{IP-T}$ was reduced to 70 bar, as well as the pressure after the LP steam turbine 2 ($p_{CD}$). However, the attempt to increase the exergetic efficiency was not successful, see Table 6.

Decreasing the TIT and the $\pi_{CM}$ would decrease the material costs as well as the gas turbine output capacity, which would be reflected in a reduction in its investment cost. Nonetheless, both factors were not taken into consideration as the selected turbine has a particular output capacity and operating temperature range that could not be modified.

The components’ obtained results of the first iteration are shown in Table 6. After the first iteration, the cost rate $({Ċ}_{D,k}+$ ${Ż}_{k})$ of the solar field, the combustion chamber, and the air-cooled condenser were successfully reduced compared with the base design. However, the efficiency of the LP steam turbine 2 decreased by 0.1%, although the objective was to increase it. No possible investment cost reduction in any of the gas turbine components is feasible, as it is based on standard commercially available technologies.

The first iteration resulted in a reduced LCOE of 37.7 $/MWh and 41.1 $/MWh for day and night operations, respectively, with a weighted average of 39.6 $/MWh. The specific investment cost was reduced to 1089 $/kW_ex_. The system exergetic efficiency increased during day and night operations to 51.5% and 59.0%, respectively.

### 4.3. Second Iteration

In the second iteration, parametric optimization was applied as in the previous iteration. The applied changes according to the results obtained in the first iteration as well as the new operating parameters of the second iteration are given in Table 7.

Aiming to reach further reduction in the LCOE, and after positively achieving the objectives of the solar field, the combustion chamber, and the air-cooled condenser, their operating parameters were further changed in the same manner as in the first iteration.

The mass flow rate inside the LFC was further reduced to 73.1 kg/s, increasing the steam temperature to 550 °C, and the pressure was further increased to 60 bar, thus resulting in an exergetic efficiency of 42.1%.

For the combustion chamber, a further increase in the $\pi_{comp}$ to 22.1 and the TIT to 1400 °C led to an increase in the exergetic efficiency to 72.4% and reduction in the ${Ė}_{D}$ from 226.3 MW to 219.5 MW.

The further reduction of $p_{CD}$ in the second iteration resulted in a lower condensation temperature of 39 °C, thus decreasing the amount of air needed for condensation to 18.2 ton/s. The condenser investment cost was reduced from $48.2 million initially to $46.7 million. For achieving an increased exergetic efficiency of the LP steam turbine 2, unlike the first iteration, the $p_{IP-T}$ was increased to 90 bars instead of decreasing it in the first iteration from 80 bar to 70 bar. However, an increase in the exergetic efficiency was also not successful.

For the gas turbine expander and compressor, the approach of optimization was changed to an increase in the exergetic efficiency instead of a reduction in their investment cost. However, recommendations for achieving such a result through decreasing the $\pi_{comp}$ and TIT were not applied owing to their higher influence over the combustion chamber, as can be seen in Table 7.

Table 8 shows the results on component level obtained in the second iteration. After the second iteration, the cost rate $({Ċ}_{D,k}+$ ${Ż}_{k})$ of the solar field and the combustion chamber and the air cooled condenser was further reduced. Although the pressure of the intermediate-stage turbine was increased in contrast to the first iteration, the efficiency of the LP steam turbine 2 also decreased by the same percentage of 0.1%. A slight decrease in the cost rate of the expander and compressor is indirectly attained through optimizing the parameters of the combustion chamber ($\pi_{comp}$, TIT).

**Figure 3:**
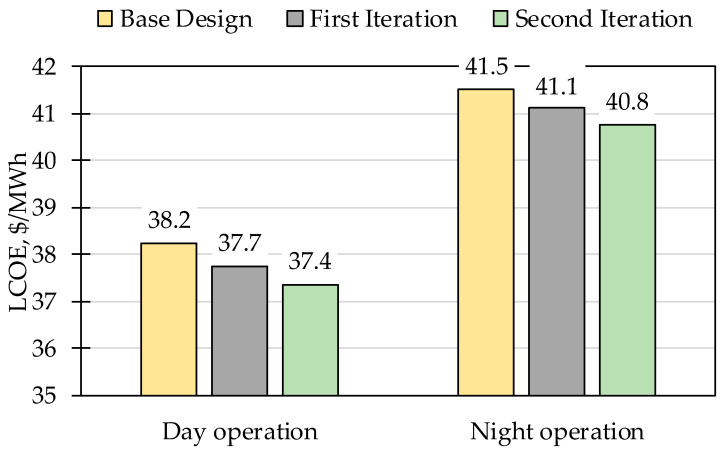
Levelized cost of electricity (LCOE) comparison between iterations for day and night operations.

**Figure 4:**
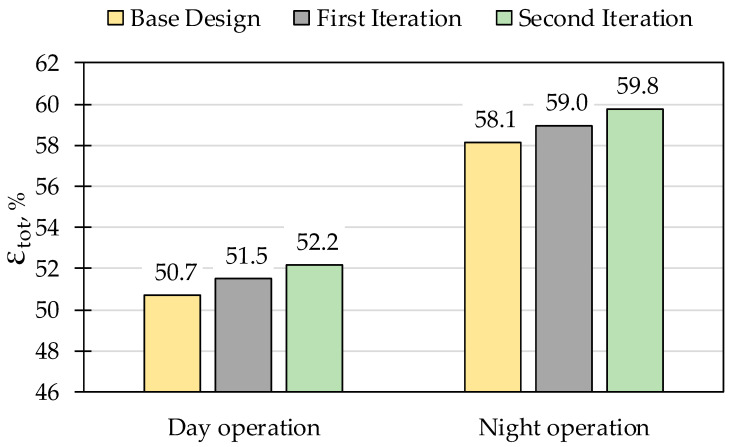
Exergetic efficiency comparison between iterations for day and night operations.

The results of all iterations for both day and night operations on the system level are compared in Figure 3 and Figure 4. In the second iteration, a further decrease in the LCOE to 37.4 $/MWh and 40.8 $/MWh during day and night operations, respectively, was achieved having a weighted average of 39.2 $/MWh. Moreover, the systems’ exergetic efficiencies were increased during both day and night operations to 52.2% and 59.8%, respectively. Such modifications reflected a slight decrease in the specific investment cost per installed capacity to 1088 $/kW_ex_.

Throughout the iterations, the LCOE and exergetic efficiency of the system were improved. The exergoeconomic optimization resulted in a decrease of 0.8 $/MWh and 0.7 $/MWh for day and night operations, respectively, as seen in Figure 3. The exergetic efficiency of the ISCC was also improved throughout the iterations, increasing by 1.5% and 1.7% during day and night operations, respectively (Figure 4). The specific investment cost of the ISCC was slightly affected, which was reduced by 3 $/kW_ex_ throughout the iterations.

### 4.4. Discussion and Validation

After the optimization was conducted, the results were verified by comparing them with similar existing integrated solar combined-cycle and CSP plants. In Table 9, the results for the total capital investment (TCI) and specific investment cost per kW installed capacity of the proposed ISCC system after optimization are compared to four ISCC systems that started operation in 2010, 2011, 2011, and 2018 in Morocco, Egypt, Algeria, and Saudi Arabia, respectively.

The installed capacity of the proposed system is most similar to the Ain Beni Mathar project (470 MW), reaching 21% higher specific investment cost of 1319 $_2018_/kW, and the Yazd project (467 MW), reaching 4% lower specific investment cost of 1047 $_2018_/kW. The relatively close specific investment cost may be attributed to the similar capacities.

The two ISCC plants, Kuryamat and Hassi R’mel, were reported to have a significantly higher specific investment cost of 2788–3069 $_2018_/kW. This may be explained by the smaller scale of the systems of 140–150 MW, as well as the choice of CSP technology. All of the operating ISCC systems listed in Table 9 operate with parabolic trough collectors and a secondary heat transfer media (CSP with HTF), while the proposed design employs linear Fresnel collectors with direct steam generation (LFC with DSG). The cost data for ISCC plants utilizing LFC and DSG were unfortunately not available (Dadri project, Table 1).

The LFC technology coupled with DSG is believed to have economic advantages over PTC with HTF. For pure CSP systems utilizing parabolic trough collector technology, the total installed plant costs (without storage) ranged from 2710 to 11,975 $/kW_2018_ (1984–2016); the majority of projects were in the range of 6380–9570 $/kW_2018_ [54]. PTC-based CSP plant costs are significantly higher than the costs for anticipated LFC projects. Linear Fresnel direct steam systems without storage projected specific costs are 2940–3675 $/kW_2018_ [55], based on inputs from the relevant industry. Moreover, reviewed CSP plants of similar size with start of operation (2013–2014) show a significant difference in costs between both technologies.

The Dhursar CSP project in India (125 MW), for instance, was reported to have an investment cost of $400 million [56], while the Shams 1 CSP plant in United Arab Emirates (100 MW) required an investment of $600 million [57]. The Dhursar plant operating with linear Fresnel collectors with direct steam generation had a 47% lower specific investment cost (approx. 2930 $_2018_/kW) than the Shams 1 plant, which is based on PTC technology with HTF (5094 $_2018_/kW). This is why, despite the lower solar share of the ISCC plants shown in Table 9, the utilization of PTC may explain the higher specific investment costs of operating ISCC plants (1319–3069 $_2018_/kW) in comparison with the proposed design. Although the LFC is cheaper than the PTC used in the Yazd project, the slightly higher cost of the proposed ISCC in comparison with Yazd may be attributed to the increased solar share (by 13 percentage points).

Waad Al Shamal project is hardly comparable to the proposed design (475 MW) owing to its significantly larger capacity of 1.39 GW. The low specific investment costs of only 785 $_2018_/kW can be justified by the economy of scale, both CCPP and CSP specific component costs decrease with size. Another reason for the drop in the costs could be the learning rate of the CSP technology. A drop in CSP costs of 79% is predicted until 2022 with reference to the costs in 2010 [58].

Finally, despite the fact that only a few ISCC plants have been built thus far and cost comparison is rather difficult, the results obtained in the presented analysis could be justified. Moreover, the potential for cost reduction through the utilization of LFC and DSG technology, as well as the suggested system design and parameters identified in the optimization, were shown.

## 5. Conclusions

In this research, a novel natural gas-fired integrated solar combined-cycle power plant was proposed and simulated under day and night operation conditions. The plant utilizes linear Fresnel collectors, which offer a cheaper alternative of collecting solar thermal energy and are a mature technology for employing direct steam generation. Direct steam generation was shown to reduce the power plant configuration complexity and to reach higher temperatures that are difficult to achieve using synthetic oil, achieving higher overall plant efficiency, apart from being more environmentally friendly.

The power plant was analysed and evaluated with the aid of exergy-based methods. Analysis of the base case resulted in a weighted average LCOE of 40.0 $/MWh and an exergetic efficiency of 50.7% and 58.1% for day and night operations, respectively. Applying the exergoeconomic iterative optimization, the LCOE was successfully reduced to weighted average of 39.2 $/MWh, while increasing the system exergetic efficiency during both operations to 52.2% and 59.8%, respectively. The system specific investment cost was slightly affected by the optimization, which was reduced from 1091 $/kW_ex_ to 1088 $/kW_ex_.

The specific investment cost of the proposed plant was verified with and compared to existing similar plants in the MENA region. For plants with similar capacity, the results obtained were shown to be relatively comparable. Owing to employing the less costly CSP technology, the proposed system enables a 13% larger solar share and competitive costs. Consequently, in particular with the proposed technology and configuration, integrated solar combined-cycle plants were proven to facilitate and ensure the transition towards higher shares of renewable power without large economical burdens especially for country like Egypt, as a case study for the MENA region, where natural gas resources already exist.

## Figures and Tables

**Table 1:** Selected integrated solar combined-cycle (ISCC) power plants. PTC, parabolic trough collector; LFC, linear Fresnel collector.

	Plant Name	Country	Technology	Capacity [MW]	Solar Share [MW]	Starting Date	Source
1	Ain Beni Mathar	Morocco	PTC	470	20	October 2010	[17]
2	Hassi R’mel	Algeria	PTC	150	20	July 2011	[11]
3	Kuraymat	Egypt	PTC	140	20	June 2011	[17]
4	Martin Next Generation Solar Energy Center	USA	PTC	3780	75	December 2010	[11]
5	Yazd	Iran	PTC	467	17	August 2010	[18]
6	Duba 1	Saudi Arabia	PTC	550	43	UC ^1^	[19]
7	Waad Al Shamal	Saudi Arabia	PTC	1390	50	July 2018	[19,20]
8	Dadri	India	LFC	1820	14	UC ^1^	[19,21]

^1^ Under construction.

**Table 2:** Technical data for simulation. ACC, air-cooled condenser; DNI, direct normal irradiance; LHV, lower heating value.

	Unit	Night	Day		Unit	Night	Day
General				Solar field			
Capacity	MW	475		Peak optical efficiency	%	68	
Humidity	%	34.8		Design point DNI	W/m^2^	-	850
Ambient temp.	°C	13	27.4	Steam temp.	°C	-	500
Fuel LHV	kJ/kg	50,015		Outlet pressure	bar	-	50
Fuel flow rate	kg/s	15.8	13.7	Collector pressure loss	bar	-	10
Exhaust gases temp.	°C	106	98	Solar share	%	-	17
Gas turbine system				Steam cycle			
Exp. isentropic eff.	%	92		Turbines isentropic eff.	%	88	
Comp. isentropic eff.	%	90		High pressure	bar	170	156.8
Compression ratio	-	20.0:1	18.0:1	Intermediate pressure	bar	80	74
Air to fuel ratio	-	2.516		Low pressure	bar	9.5	15
Turbine inlet temp.	°C	1334	1287	ACC pressure	bar	0.097	
Turbine outlet temp.	°C	600		ACC exhaust temp.	°C	40	

**Table 3:** Components’ ranking according to the highest total cost rates. LP, low-pressure.

Rank	Component	$\varepsilon_{k}$ [%]	$f_{k}$ [-]	${Ċ}_{D,k}+$${Ż}_{k}$ [$/h]
1	Solar Field	41.3	0.413	8251
2	Combustion Chamber	71.5	0.030	3530
3	Expander	96.4	0.660	1475
4	Air Cooled Condenser	N.A.^1^	0.808	1414
5	Compressor	95.1	0.601	912
6	LP Steam Turbine 2	88.4	0.329	829

^1^ Dissipative component.

**Table 4:** Identification of decision variables.

Component	Decision Variables
Solar Field	${\dot{m}}_{SF}$, $p_{SF}$
Combustion Chamber	$\pi_{\mathrm{CM}}$, TIT
Expander	$\pi_{\mathrm{CM}}$, TIT
Air Cooled Condenser	$p_{SF}$, $p_{CD}$
Compressor	$\pi_{\mathrm{CM}}$
LP Steam Turbine 2	${\dot{m}}_{SF}$, $p_{SF}$, $p_{IP-T}$, $p_{CD}$

${\dot{m}}_{SF}$ solar field mass flow rate; $p_{SF}$ solar field pressure; $\pi_{\mathrm{CM}}$ compressor pressure ratio; TIT gas turbine inlet temperature; $p_{CD}$ condenser pressure; $p_{IP-T}$ intermediate-stage steam turbine pressure.

**Table 5:** Suggested changes of the base case system parameters for the first iteration.

Rank	Component	${Ċ}_{D,k}+{Ż}_{k}$ [$/h]	f [-]	Objective	${\dot{m}}_{SF}$	$p_{SF}$	$\pi_{CM}$	TIT	$p_{IP-T}$	$p_{CD}$
				${Ż}_{k}$ $↑\;\mathrm{or}\;\varepsilon_{k}↓$						
1	Solar-Field	8251	0.413	$\varepsilon_{k}$ ↑	↓	↑	-	-	-	-
2	GT-CC	3530	0.030	$\varepsilon_{k}$ ↑	-	-	↑	↑	-	-
3	GT-EXP	1475	0.660	${Ż}_{k}$ ↓	-	-	↓	↓	-	-
4	ACC	1414	0.808	${Ż}_{k}$ ↓	-	↑	-	-	-	↓
5	GT-COMP	912	0.601	${Ż}_{k}$ ↓	-	-	↓	-	-	-
6	LP-TUR-2	829	0.329	$\varepsilon_{k}$ ↑	↓	↓	-	(↑)	↓	↓
				${Ċ}_{D,k}+$${Ż}_{k}$↑	0	9665	3530	3530	0	0
				${Ċ}_{D,k}+$${Ż}_{k}$↓	9080	829	2387	1475	829	2243
				Suggestion	↓	↑	↑	↑	↓	↓
				Initial Values	77.1 kg/s	50 bar	20.1	1334°C	80 bar	0.097 bar
				New Values	75.1 kg/s	55 bar	21.1	1365 °C	70 bar	0.08 bar

**Table 6:** First iteration obtained results of selected components.

Rank	Component	$\varepsilon_{k}$ [%]	$\;f_{k}$ [-]	${Ċ}_{D,k}+{Ż}_{k}$ [$/h]
1	Solar Field	41.8	0.418	8167
2	Combustion Chamber	71.9	0.031	3429
3	Expander	96.4	0.667	1459
4	Air Cooled Condenser	N.A.	0.837	1343
5	Compressor	95.2	0.612	897
6	LP Steam Turbine 2	88.3	0.331	837

**Table 7:** Second iteration suggested changes.

Rank	Component	${Ċ}_{D,k}+$${Ż}_{k}$ [$/h]	f [-]	Objective	${\dot{m}}_{SF}$	$p_{SF}$	$\pi_{CM}$	TIT	$p_{IP-T}$	$p_{CD}$
				${Ż}_{k}$ $↑\;\mathrm{or}\;\varepsilon_{k}↓\;$						
1	Solar-Field	8167	0.418	$\varepsilon_{k}$ ↑	↓	↑	-	-	-	-
2	GT-CC	3429	0.031	$\varepsilon_{k}$ ↑	-	-	↑	↑	-	-
3	GT-EXP	1459	0.667	$\varepsilon_{k}$ ↑	-	-	↓	↓	-	-
4	ACC	1343	0.837	${Ż}_{k}$ ↓	-	↑	-	-	-	↓
5	GT-COMP	897	0.612	$\varepsilon_{k}$ ↑	-	-	↓	-	-	-
6	LP-TUR-2	837	0.331	$\varepsilon_{k}$ ↑	↓	↓	-	(↑)	↑	↓
				${Ċ}_{D,k}+$${Ż}_{k}$↑	0	9510	3429	3429	837	0
				${Ċ}_{D,k}+$${Ż}_{k}$↓	9004	837	2356	1459	0	2180
				Suggestion	↓	↑	↑	↑	↑	↓
				Initial Values	75.1 kg/s	55 bar	21.1	1365 °C	70 bar	0.08 bar
				New Values	73.1 kg/s	60 bar	22.1	1400 °C	90 bar	0.07 bar

**Table 8:** Second iteration obtained results of selected components.

Rank	Component	$\varepsilon_{k}$ [%]	$\;f_{k}$ [-]	${Ċ}_{D,k}+$${Ż}_{k}$ [$/h]
1	Solar Field	42.1	0.421	8096
2	Combustion Chamber	72.4	0.032	3328
3	Expander	96.4	0.673	1445
4	Air Cooled Condenser	N.A.	0.854	1297
5	Compressor	95.2	0.621	884
6	LP Steam Turbine 2	88.2	0.330	834

**Table 9:** Plant characteristics of the proposed design and three ISCC projects in the MENA region. DSG, direct steam generation; HTF, heat transfer fluid; TCI, total capital investment.

Project	Proposed Design	Ain Beni Mathar	Yazd	Kuryamat	Hassi R’mel	Waad Al Shamal
Technology	DSG- LFC	HTF-PTC	HTF-PTC	HTF-PTC	HTF-PTC	HTF-PTC
Country	Egypt	Morocco	Iran	Egypt	Algeria	Saudi Arabia
Investment year (approx.)	2020	2007	2007	2007	2007	2016
TCI, Mio $	517	540	426	340	401	980
Spec. Investment, $_2018_/kW	1088	1319	1047	2788	3069	785
Capacity, MW_el_	475	470	467	140	150	1390
Solar share, MW_el_	81	20	17	20	20	50
Solar share, %	17	4	4	14	13	4
Source	-	[17]	[18,51]	[17,52]	[11,51]	[20,53]

## Abbreviations

ACC	Air cooled condenser
AFUDC	Allowance for funds used during construction
CC	Carrying charges
CCPP	Combined-cycle power plant
CELF	Constant escalation levelization factor
CI	Capital investment
COMP	Compressor
COND	Condenser
CRF	Capital recovery factor
CSP	Concentrated solar power
DSG	Direct steam generation
EXP	Expander
FC	Fuel cost
FCI	Fixed capital investment
GT	Gas turbine
HP	High pressure
HRSG	Heat recovery steam generation
HTF	Heat transfer fluid
IP	Intermediate pressure
ISCC	Integrated solar combined-cycle
LCOE	Levelized cost of electricity
LFC	Linear Fresnel collector
LHV	Lower heating value
LP	Low pressure
NG	Natural gas
OM	Operation and maintenance
OMC	Operation and maintenance cost
PTC	Parabolic trough collector
TCI	Total capital investment
TIT	Turbine inlet temperature
TRR	Total revenue required
TUR	Turbine
Nomenclature	
c	Specific cost per unit exergy
Ċ	Cost rate
e	Specific exergy
Ė	Exergy rate
f	Exergoeconomic factor
*h*	Specific enthalpy
$\dot{m}$	Mass flow rate
P	Pressure
Q	Thermal energy
r	Relative cost difference
*s*	Entropy
*T*	Temperature
Ż	Component cost rate
Greek symbols	
λ	Air to fuel ratio
τ	Operation time
ε	Exergetic efficiency
π	Compression ratio
subscripts	
*D*	Destruction
F, f	Fuel
j	Stream, state
k	k-th component
L	Levelized, losses
P	Product
sol	Solar
*tot*	Total
0	Reference
superscripts	
PH	Physical
CH	Chemical
